# The experiences of practitioners working with young people exposed to online sexual abuse

**DOI:** 10.3389/fpsyt.2023.1089888

**Published:** 2023-03-13

**Authors:** Ethel Quayle, Matthias Schwannauer, Filippo Varese, Kim Cartwright, William Hewins, Cindy Chan, Alice Newton, Prathiba Chitsabesan, Cathy Richards, Sandra Bucci

**Affiliations:** ^1^Department of Clinical and Health Psychology, School of Health in Social Science, University of Edinburgh, Edinburgh, United Kingdom; ^2^NHS Lothian, University of Edinburgh, Edinburgh, United Kingdom; ^3^Division of Psychology and Mental Health, School of Health Sciences, Faculty of Biology, Medicine and Health, The University of Manchester, Manchester, United Kingdom; ^4^Greater Manchester Mental Health NHS Foundation Trust, Manchester, United Kingdom; ^5^Pennine Care NHS Foundation Trust, Ashton-under-Lyne, United Kingdom; ^6^Division of Psychology and Mental Health, The University of Manchester, Manchester, United Kingdom

**Keywords:** Online Child Sexual Abuse, child and adolescent psychiatric care, risk assessment, routine practice, qualitative

## Abstract

**Introduction:**

This qualitative study explored healthcare professionals' current understanding of, and clinical practices related to, Online Child Sexual Abuse (OCSA).

**Methods:**

Data were collected across two UK sites (Manchester and Edinburgh). Interviews and one focus group were held with 25 practitioners working in services offering clinical support to young people who have experienced OCSA. Thematic analysis of the data identified three overarching themes and 10 subthemes related to the research questions: (1) the breadth of the problem; (2) working with OCSA; and (3) the emotionally charged nature of OCSA.

**Results:**

While practitioners recognized OCSA as problematic, they differed in how they conceptualized it. There was a heightened awareness of the role that sexual images played in OCSA and concerns about first-person-produced imagery by Children and Young People (CYP). Practitioners described a generational gap related to their technology use and that of the young people they worked with. Practitioners also described a paucity of referral pathways and concerns that there was no training available to them. Organizational barriers meant that questions about technology use were not routinely included in assessments and often there was reliance on young people making disclosures.

**Discussion:**

Novel findings from this study were the psychological impacts that such cases had on practitioners, which may indicate a need for organizational support for staff as well as further training needs. Existing frameworks that help conceptualize and assess the role of technology as part of the ecology of the child may have great utility for practitioners.

## Introduction

Children and Young People (CYP) increasingly lead lives interwoven with technology use where online and offline worlds are entwined and simultaneous (1). The EU Kids Online research group (2) showed that in the 19 European countries surveyed, for most CYP smartphones are now the preferred means of going online, with the majority reporting using devices “daily” or “almost all of the time.” Similarly, in the US, almost 95% of CYP aged 13–17 have access to smartphones, with 45% saying they use smartphones “almost constantly” (3). In 2020, nearly all UK children aged 5–15 went online, with just over half of 12–15-year-olds having negative experiences, most commonly being contacted by someone they did not know who wanted to be their friend (4). One function of smartphones (and also tablets) is the ability to take photos and videos which makes it possible for people of all ages to create and share digital artifacts that relate to all aspects of everyday life (5). Increasingly, this includes sexual lives where digital media have transformed how we initiate, maintain, and terminate our intimate relationships (6). A recent review found that sharing self-made sexually explicit images is a modern form of sexual communication and increasingly used within dating and romantic relationships by adults and adolescents (7) using technology platforms that have changed over time (8). The availability of smartphones has meant a move to applications (apps) such as Snapchat and WhatsApp to facilitate sharing of sexually explicit content and making it more difficult to detect.

Creating “selfies” (often co-constructed with others) is ubiquitous across all generations and contributes to a CYP's digital identity (9). “Sexy selfies” are often positioned as problematic for (but not necessarily by) CYP (10) and seen as increasing the risk of online victimization (11) through the coercive production of sexual images as well as the identification of potential victims (12, 13). Digital technologies are embedded in our everyday practices and form an intrinsic part of private and public experiences (14). This has led to an argument for thinking of a “cybersystem” as part of the ecology of the child (15–17). Such a conceptualization (18) might help explain how the environment, including the online environment, can perpetuate and escalate the harms that follow from Online Child Sexual Abuse (OCSA) and increases the likelihood that practitioners working with children and adolescents will address the involvement of technology in their lives. The internet has created not only opportunities for CYP to act as receivers, participants, and actors in the digital world, but has also created spaces of social interaction which hold potential for exposure to online risks, including sexual risks such as abuse and exploitation. It has been suggested that the internet does not make children more vulnerable but might make already vulnerable children more accessible, and there is a strong argument for reconfiguring existing violence research frameworks to include online violence if we are to prevent and manage it (19, 20).

Online Child Sexual Abuse, or technology-facilitated abuse (21), refers to situations involving digital, Internet and Communication Technologies (ICT) at some point during the abuse or exploitation. Furthermore, a review of survey, conviction, and image-related data concluded that there was converging evidence of an increase in some forms of OCSA (22, 23). This includes a spectrum of abusive and harmful practices, such as the production, possession, or sharing of Child Sexual Abuse Material (CSAM), live streaming of sexual abuse and online grooming of children for sexual purposes, the sharing of self-produced sexual content involving minors, sexual harassment, and unwanted exposure of a minor to sexual content. Increasingly, many of the sexual images associated with OCSA are First Person Produced Images (FPPI), also called “sexts” (24), which are largely created by CYP during online sexual grooming (25, 26) or forwarded non-consensually by peers or adults.

In recent years, there has been growing research interest in the experiences and impact of OCSA on CYP. Children and Young People who have been abused through Child Sexual Abuse Material (CSAM) production appear to experience additional problems which are different to those associated with contact abuse. There are two prominent features: (i) anxiety about whether the victim might be seen as a willing participant in the abuse; and (ii) fear of being recognized if someone they knew saw the images (27, 28). These concerns are often accompanied by feelings of guilt and shame and an ongoing sense of vulnerability (29). Self-blame and self-criticism have been identified in other studies (21, 30) alongside feelings of being enmeshed or controlled within a relationship. The psychological consequences for CYP may also include depression, self-harm, anxiety, post-traumatic stress disorder (PTSD) symptoms, perceived identity changes, and loss of epistemic trust (31, 32). This in turn reduces the likelihood of disclosing or reporting the abuse. However, importantly, there is often a limited understanding by professionals of the risks associated with OCSA and the possible consequences for victims (33). This may potentially lead to CYP remaining at risk, with professionals failing to offer protection on referral or appropriate support.

Early work in the field of OCSA revealed a common consensus over key aspects that differentiate online victims from children abused and exploited offline. These are linked to a perceived lack of control over the disclosure process; feelings of shame and complicity; inability to find closure due to the persistent existence of images; and a potential for re-traumatization or re-victimization when confronting the evidence. As already noted, sexual images are a feature of much of this abuse and once identified they become the “permanent products” of offense behavior. An analysis of cases concerning identified children who were abused and photographed between 1991 and 1994 noted that most of the children did not disclose, and even when confronted with the photographic evidence of the abuse, would only acknowledge what they thought was already known (34). These authors (also clinicians) described these cases as: children who cannot tell; children who do not tell; the traumatic crisis; memory loss; and active attempts to forget.

This early work resonates with more contemporary studies (35). One study analyzed 52 cases of adolescents who arrived at a Child Advocacy Center (CAC) following OCSA and looked at the multi-faceted nature of disclosure, including the experiences of parents who wanted simply to bring the subject to a close. The difficulty in disclosing such incidents was illustrated by the fact that 20 children in this sample were reluctant to collaborate during the CAC process, and most of the incidents were revealed following a police investigation rather than by a disclosure initiated by the children themselves. These authors concluded that there needs to be a better understanding of what it means for CYP subjected to OCSA to disclose as this may improve and help modify future prevention and intervention efforts in this area.

An unpublished systematic review by the Marie Collins Foundation (MCF) (36) of the recovery needs of young people exposed to OCSA revealed an emerging field where there was little academic engagement. Identified studies focused on the challenges professionals faced when responding to children abused and exploited online (37) with a general agreement amongst practitioners, including social workers and other mental health professionals, that OCSA cases are extremely challenging and poorly understood (38). In terms of “best practice” care available to victims, most children gained access to therapeutic help through youth welfare services, other public services, or the police. Professionals offered care largely based on trauma treatment models with an aim to stabilize victims and to facilitate disclosure and recovery (37) or help young people to manage post-trauma symptoms.

At present there is little empirical evidence or evidence-based guidelines about how professionals might respond to victims of OCSA. One Canadian study highlights how practitioners differ in their conceptualization of what constitutes OCSA, their levels of concern about the issue, and their understanding of the potential effects on the child (39). In this study, respondents acknowledged that in the absence of clinical guidance, conceptualizations are shaped by social discourse and anecdotal reports of cases of “conventional” child abuse in which the victim was photographed. Professionals were unsure what questions to ask about online aspects of the abuse and they frequently lacked confidence about the appropriateness of diagnostic assessments, and rarely considered addressing the issue directly with young people.

More recently, a Canadian study of CAC staff indicated that while most practitioners dealt with OCSA cases (grooming, luring, sexual abuse, and child sexual abuse imagery), they were more likely to encounter problems with these cases than CSA cases and yet would use similar approaches with young people regardless of whether technology had been involved or not (40). The same research group completed a parallel study with mental health providers in Canada which resulted in similar findings. The authors concluded that there was a need for improvement in practitioners understanding of how technology is used to exploit CYP (41).

Recent research in the UK has noted that within Child and Adolescent Mental Health Services (CAMHS), staff are not always aware of the current research findings associated with OCSA or the guidance available around digital safety (42). The authors of this research used a mixed-methods design with clinicians working in CAMHS (of which 12 took part in interviews), clinicians expressed awareness of, and concerns around, a number of digital risk issues, but there were gaps in their knowledge and practice. Different factors influenced whether clinicians inquired about OCSA. This included lack of confidence about their knowledge and skills, few opportunities to engage due to, for example, a lack of resources, and motivation to change routine ways of practicing. A further UK study explored how local services working with CYP (social care, health, police), managed cases of OCSA (33). There was awareness of OCSA, but the focus was narrow such that people only identified particular types of abuse (such as sharing FPPI) which led to services not exploring wider online risks or their antecedents. As with the UK CAMHS study (42), assessment tools were generic and tended to omit online risks unless specific safeguarding issues were raised. Furthermore, multi-agency collaboration was problematic as there was an absence of referral pathways and staff had few opportunities for specific training related to online risks. This study was replicated with a larger sample of 29 practitioners which similarly showed that practitioners demonstrated a limited and fragmented understanding of young people's online vulnerabilities and risks, with no integration of these issues into routine practice. The emphasis for practitioners was on identifying risk rather than understanding the experiences of young people, and there were no appropriate training, or assessment tools available to them (43). One response to a lack of training for practitioners has been the development of a short course designed to increase competencies and confidence in responding to the therapeutic needs of young people following online abuse (44).

## The research questions

This qualitative research explored healthcare professionals' current understanding of, and clinical practices related to, OCSA.

## 3. Materials and methods

### 3.1. Participants

Participants (*N* = 25) were selected across two sites (Manchester, Edinburgh) and were recruited through National Health Service (NHS) Child and Adolescent Services (CAMHS), a Sexual Assault Referral Center (SARC), and Kooth (a NHS commissioned mental health e-therapy provider). Inclusion criteria were healthcare professionals currently working in CAMHS, SARC, or Kooth with a good understanding of the English language. The sample size was determined by paying attention to the study aims and in-depth exploration of a sample that has shared characteristics (45, 46).

### 3.2. Design

We utilized a qualitative study design. Individual interviews (Manchester, Edinburgh) and a focus group (Edinburgh) were used to provide a range of accounts across healthcare practitioners. Accounts were analyzed using Reflexive Thematic Analysis (RTA) (47, 48). Reflexive Thematic Analysis is a theoretically flexible interpretive approach to qualitative data analysis that facilitates the identification of patterns and themes within the data, but where the researcher plays an active role in knowledge production. While there is no expectation that the codes or themes identified by one researcher will be reproduced by another, it is accepted that multiple coders may be beneficial to sense check ideas or explore different interpretations of the data (49). In this respect, coding was examined by, and discussed with, members of the team.

The interview and focus group questions addressed practitioners' understanding of CSA and how it was routinely assessed and managed therapeutically. In addition, beliefs about the strengths and challenges of a digital app, and expectations about the app and its use in practice were also included but these are not addressed within this publication. The interviews were semi-structured, and questions were open-ended with sequencing dictated by the flow of the exchange. Probes were used to aid further elaboration on the responses.

### 3.3. Procedure

The relevant Institutional and Health Research Authority ethics approvals were granted (REC Number: 21/WS/0160) and the protocol was registered at ClinicalTrials.gov (ClinicalTrials.gov Identifier: NCT05006053). Participants were approached through service managers of the relevant NHS Trusts and e-therapy provider platform to ask for permission for an advert/flier to be circulated to staff via email, their website, social media, newsletters, weekly bulletins, and announcements. Researchers also attended departmental meetings where interested staff were encouraged to email the researchers for information. In addition, an advert was circulated via our research group's website and social media account. People who confirmed that they would like to take part in the study were given the choice to meet online individually or as part of a focus group. Consent was obtained verbally (either online or by phone) using an oral consent script. Interviews lasted approximately 1 h and were transcribed verbatim, anonymised, and given an identifying code (also used to identify where extracts came from within the Results) and stored securely. Field notes and reflexive logs were kept throughout. Participants completed a brief demographic form once consent had been obtained and prior to the interview or focus group.

### 3.4. Data analysis

Interviews were audio-recorded and then transcribed verbatim. Analysis was supported by the end-to end encrypted software application Dedoose for qualitative and mixed methods research (50). A predominantly inductive approach was adopted. Data was open-coded and meanings based on the interpretations made by respondents was emphasized. However, the questions asked in the interview, although used flexibly, meant that deductive analysis was also employed to ensure that the open coding allowed for the identification of themes that were meaningful to the research questions posed. Therefore, both semantic and latent codes were used and we followed the proposed recursive and iterative six-stage analytical process to facilitate coding and theme-identification: (i) familiarization with the data; (ii) generating initial codes; (iii) generating themes; (iv) reviewing potential themes; (v) defining and naming themes, and (vi) producing the report (47). The codes were primarily developed by EQ and SB and were further sense checked by WH and reviewed by members of the research team as they were developed. Second coding was not used and there was no attempt to determine inter-rater reliability (51). Instead, quality assurance of the coding, theme development and the final write up were guided by a recent tool for evaluating research quality (52). This tool consists of 20 questions to guide the assessment of RTA research methodology which focus on a justification of the method and methodology and a well-developed and coherent analysis.

## Results

A total of 25 professionals were recruited across two sites (Manchester and Edinburgh). Six of the Edinburgh sample opted to be part of a focus group. A summary of participant characteristics is presented in Table 1.

**Table 1:** Summary of participant characteristics.

**Overall sample**	***N* (%)**
**Gender**	
Female	22 (88)
Male	03 (12)
**Ethnicity**	
White British	19 (76)
White (any other background)	04 (16)
Asian	01 (04)
European	01 (04)
**Area of service**	
CAMHS	21 (84)
SARC	02 (08)
E-therapy	02 (08)
Mean (range) years working for service	13 (1–37)

Thematic analysis of interview and focus group data identified three overarching themes and ten subthemes related to the research questions: (1) the breadth of the problem; (2) working with OCSA; and (3) the emotionally charged nature of OCSA (Table 2).

**Table 2:** Themes and sub-themes.

**Themes**	**Subthemes**
The breadth of the problem	A fragmented understanding
	A spectrum of abuse involving technology
	Peer-related activity
Working with OCSA	Disclosure rather than assessment
	Organizational barriers to knowing
	A question of trust
	Something new to grapple with
It's emotionally charged	Impact on the practitioner
	Reflecting on own experiences
	Involving caregivers

###  Theme 1: The breadth of the problem

#### Subtheme 1: A fragmented understanding

Across all respondents, there was an awareness of OCSA but also a struggle to articulate what this might include. This resulted in naming specific categories of abusive practices rather than a consideration of how technology may play a part across the wider spectrum. The category most often referenced was “online grooming,” but respondents also talked of non-consensual sharing of sexual images, sexual harassment, pornography exposure, and sexualized behavior. Within each of these there were tensions around what was included in the category; for example, whether online grooming should include an intention to meet the young person by the perpetrator, or whether grooming may take place if there was no physical contact:

“*Erm, for me it includes the, the contact, erm, the grooming, erm, whether that's someone seeking out contact with a young person, erm, well it could be that it starts off as friendship and then sexual abuse, erm, comes along, erm, at a later time, whether that was, er, the intent of the initial contact or not, it, it would be possible, but also includes people seeking out young people for that, erm, for that purpose, to sexually abuse them, to groom them, ask for pictures, erm, arrange to meet up for physical, erm, physical contact and a sort of abuse, erm, but I think, online sexual abuse includes, where there's been no physical meet up, as well” (MAN-016)*.

Frequent reference was also made to OCSA as sharing of nude or sexual pictures and videos which could take place between a young person and an adult or between peers. In this context, such sharing was seen as abusive whether or not (in the case of peers) the activity appeared consensual:

“*Well I think it, for me, it can be as, erm, simple as somebody sending naked pictures or asking for someone to take pictures on phone or online” (ED-009)*.

Whether sharing sexual images constituted OCSA was not always easy to determine and there was not always consensus about when some activities would be seen to be illegal, especially when an image was initially consensual, but then shared non-consensually:

“*Yes, erm, so this one was actually consensual between the two people, erm, the example that I'm thinking about, however, that, erm, that image was then shared which obviously was not consensual, erm, and also that young person was not aware that actually even giving their own picture to someone else or sending their own picture to someone else was, was not, was illegal, so I thought it was quite a bit of, of, erm, lack of information, …I thought there was quite a bit of misunderstanding around what, what is allowed, what's not allowed, what is illegal and what is, erm, not counting as, as illegal” (MAN-007)*.

In other cases, non-consensual sharing of images was described in the context of sexual abuse by someone known to the young person, but who was unaware that the abuse had been recorded:

“*…it's really unusual but awful case that I have worked with, where somebody was, erm, filmed whilst being abused by a parent figure and that was uploaded to the internet” (ED-FG1-006)*.

Throughout this theme, what constituted OCSA was also influenced by a consideration of the young person's agency and whether there was a shared understanding between practitioners and CYP of the appropriateness or illegality of some behaviors:

“*…kind of online abuse, takes many forms, so, I think if we kind of look at some of the research and we look at some of the kind of stories we get from young people, there are, kind of young girls almost don't bat an eyelid to having received dick pics, as it were” (MAN-009)*.

However, for most respondents what determined whether something constituted OCSA was a young person being targeted by an adult or peer where they were drawn into an exploitative relationship or activities through persuasion, flattery, or promises of love. This was the defining feature regardless of the young person's seeming compliance with the offender:

“*Yeah, and a lot of times, young people are actually totally, they completely know, you know, if it gets further, that, what that person has done is, is wrong, they know that, but then they cannot connect the dots between this person that, that they love, or that they've got this bond with and the person that did that, it, it's quite strange how they like, oh I know what he did was wrong, but he's this nice person and he's done all these good things for me and they cannot connect the dots between the two, and I think connecting those dots and being like, but actually he was doing that for that, you know” (MAN-015)*.

What was also highlighted was a perceived imbalance of power with young people targeted because they were seen as vulnerable:

“*… thinking about my own experiences of young people and the two words that popped into my head were power, and that power and differential that's there between the young person and then the alleged perpetrator, but also for me it's an exploitation of a vulnerability, that's what I sort of feel that, and I guess it feeds into power as well, that there's a recognition or an awareness that that young person, either through their age or through something else, erm, are vulnerable and there's advantage taken of that.” (ED-FG1-006)*

#### Subtheme 2: A spectrum of abuse involving technology

In contrast to using a narrow definition of OCSA, a substantial number of practitioners talked about a growing awareness of the scale and range of the problems they were encountering:

“*I mean I think my view of it has expanded since I first, you know, I think the first thing you automatically think about is, is you know pictures, videos, but, erm, it, it feels like anything that's sexually coercive or exploitative, using screens of any kind. Do you know I think, I think mobile, I think, you know whether it's PC, laptop, I think it's, erm, it covers a huge gamut of things that are, erm, abusive. Whereas I think when I first started this that I had a more narrow view of it a wee bit, but I think it's very much expanded since then, so it's using screens in any kind of sexual exploitative way, that's how I see it, harassment and things” (ED-007)*.

This expansion of what might be seen as abusive by professionals included novel forms of sexual violence such as being sent unsolicited sexual pictures, but which was also positioned by some respondents as pornography:

“*I feel like it's on such a broad range that I won't even kind of cover everything, and I think the, you know, quite, erm, I suppose subtle, like there's subtle things that perhaps may not have been considered online sexual abuse in the past that I feel should be, you know, kind of being sent unsolicited pictures” (ED-010)*.

For people who worked in services for both victims and perpetrators, there was also an agreement that OCSA covered a wide range of activities which included a potential blurring of roles when CYP's activities were also seen as potentially abuse:

“*Erm, oh it's a massive spectrum… so that can be anything from accessing material that is against the law, through to indecent images, or, erm, grooming people online, erm, I guess even accessing, er, images and sites that are prohibited as well, so we have young people who have actually accessed… the dark web, etc. er, and also young people communicating with, erm, er, pretending to be someone they're not, so er, pretending they're actually younger” (MAN-012)*.

This more inclusive definition of OCSA was also seen where practitioners focused on the use of technologies by perpetrators at any part of the offense chain:

“*Erm, abuse where, a kind of, a situation where sexual abuse is taking place and to some degree or another, online platforms have been used to, er, facilitate that happening. So it's not, not, I guess not necessarily that the incidents of abuse have happened while online but the, contact might have happened via online, or grooming might have happened online or, erm, things might have been shared online, I guess, is my impression quite a broad term” (MAN-006)*.

#### Subtheme 3: Peer-related activity

There was frequent reference to peer-related OCSA which was often accompanied by hesitancy about how this should be positioned:

“*…there is an awful lot with young people as well, erm, but I wouldn't necessarily consider those, kind of, child sexual exploitation, erm, obviously because they're both children and it's, it's kind of like, it's more peer kind of like sharing of images, erm, but they knew each other before, or they know in school and stuff, and peer on peer in school has gone mad as well” (MAN-015)*.

Such activities were seen as increasingly common in schools, when sexual images were subsequently non-consensually shared between peers which resulted in the involvement of services for young people:

“*Yeah, no, er, unsolicited images in school, that's a recurring theme… pressure in school to provide images to other people and then these images being shared within school, that's a massive thing that goes on a lot” (MAN-010)*.

Explicit reference was often made to young people being exposed to “threats to share” if they did not engage further in the exchange of sexual images, and this was also referenced in relation to abusive practices by adults:

“*Erm, yeah I was just thinking about, the children actually, erm, getting other children to share photos, erm, either of each other or something and those other children putting them online, erm, or using those photos as sort of, er, blackmail or threats, yeah” (ED-FG1-006)*.

However, the blurred boundaries between being a victim and victimizing others can be seen in the following extract where a young person was pressured into taking sexual pictures of a sibling and sharing those with an adult who was communicating with him through an online platform:

“*Images, yeah, I think it was, I don't know if it was posed or, or anything actually happening, but it was, erm, certainly naked, naked images of his younger sibling. Erm, that he facilitated I suppose, being sent to the original abuser. If that makes sense?” (MAN-016)*.

###  Theme 2: Working with OCSA

#### Subtheme 1: Disclosure rather than assessment

While there was evidence across respondents of a growing awareness of OCSA there was an acknowledgment that in many cases young people were not specifically asked about this as part of their routine assessment:

“*…but actually when I reflect again on sort of a lot of my outpatient cases, a lot of them have got trauma and online abuse as part of that trauma, but it's not something I would, with that hat on, routinely ask about. Does that make sense?” (ED-FG1-006)*.

Rather, disclosure came about indirectly during assessment where questions may have been asked about routine online activity as part of understanding a young person's presenting problems:

“*I was just thinking about it in terms of things do come up but we were asking directly about social media, what kinds of social media they use, erm, and then kind of exploring it in a little bit more detail. Or if they tend to not use social media too much, what kind of gaming, and kind of who, what kind of chat rooms, or who are their friends online and kind of going down those, almost with their, what do they spend their time doing, and who do they talk to in those online kind of arenas, erm, and sometimes felt kind of an avenue to go down” (ED-FG1-006)*.

Practitioners seemed comfortable with a more informal approach to inquiry through talking about friendships and romantic relationships rather than direct questions about online abuse:

“*I think otherwise it might come up a little bit more organically, erm, when young people are talking about relationships and they're talking about what feels safe, what feels ok to them and then, and then just sometimes the dilemmas they get into when they're in a relationship with someone and they want to do these things, but they're a bit worried about what happens if it all goes wrong to the images that have been taken” (ED-009)*.

This dependence on disclosure by the young person or through another agency did not mean that professionals were not sensitive to OCSA, and for some, there was an awareness that many young people would not be able to make a disclosure either because of a lack of trust or because the abusive relationship was important to them:

“*…but it's, we need to, if we're gonna, we need to see it from the young person's point of view and sometimes when those, those other supports aren't there, or aren't noticing anyway, then, that, how do we get the secret out, how do we pop, burst that bubble that's been built by the perpetrator, to get the support in” (MAN-016)*.

However, boundaries were often set by practitioners' concerns as to what might be appropriate questions to ask, particularly in relation to younger children:

“*…just hearing some of our younger children who go onto apps and then are encouraged to do those, erm, and then hearing about some of the, sort of, animated sites that they go onto, and that then they're directed to slightly more pornographic sort of sites, I guess I don't routinely ask that” (ED-009)*.

It was also acknowledged that some children are simply not yet ready to talk about what has happened to them:

“*So as that relationship grows that trust is developed and somebody feels able to say, oh, this happened, this, and sometimes it's that drip, drip, drip bit of, well, yeah, this happened and then I sent this and, or, or and sometimes, some occasions you kind of get, oh yeah, well I get sent pictures of somebody's dick, like however many times a day, and it's kind of a throwaway kind of comment, whereas for other people they kind of built up to say, right yeah, this and this…” (MAN-009)*.

#### Subtheme 2: Organizational barriers to knowing

While there were differences across professionals in their confidence, or the perceived need, about asking questions to young people concerning OCSA, there was apparent consensus that this was not an organizational priority:

“*No, we don't operate a diagnostic, diagnostically specified pathway, that's not the way we work. Erm, we, I guess, the, we operate in the inpatient services, we don't operate by pathway, but we might link in with, erm, community CAMHS, or social care. Sometimes social care, erm, if someone's got a social worker or early health worker might be doing some work on, for example, healthy relationships, or, erm, understanding abuse if someone's been through that, sometimes that happens” (MAN-006)*.

For one respondent it was felt to be inappropriate within their services to ask the young person direct questions about OCSA:

“*No, not, not that I've ever known, and I've never asked them an outright, ‘cos it's always been drummed into us never to just ask a leading question” (MAN-04)*.

In relation to this it was observed that there are no assessment tools available to staff:

“*But I personally am not aware of a specific assessment tool that just focuses on online sexual abuse” (MAN-01)*.

There was also a suggestion that OCSA is not necessarily perceived by services as serious as the commission of a contact offense against a child, and therefore these cases may not be prioritized:

“*…have had a few, actually, where it's been online, online, isolated, well online contact, meeting up, there's sexual assault has happened, it's been disclosed straight away, social worker will go out because of that, and say ok, everything's in place, there's no safety, safety concerns in the home and they'll wrap it up and close it. I've had a few like that, erm, and the, instead of the case, well, you know there's none, no other identifiers of child sexual exploitation, apart from the child sexual exploitation itself that's just happened, or they're seeing it as isolated online abuse, rather than a pattern” (MAN-016)*

The lack of prioritization in these cases may mean that other agencies involved in the case may not refer the young person on to children's services:

“*A lot of it starts at that, but the police don't refer as much in for support when it's just online, and images and things like that. So I think, you know, services that support don't actually know half of, half of what's going on because the police don't refer those cases in, they only refer, erm, contact cases for support” (MAN-015)*.

For CAMHS, it was also the case that while online abuse may be perceived similarly to contact abuse, decisions may be made to refer on to other non-clinical services for young people:

“*So in terms of what CAMHS would offer, I think, it would probably be a similar, yeah, I think it would be a similar approach to if someone had experienced other, I guess, erm, sexual trauma, or, erm, another kind of, erm, traumatic experience, and I think with CAMHS there's a distinction of roles that often we might, if someone said that in an assessment, that they would then go to be put on our waiting list, we often kind of link in with the wider system a bit more and think about what third sector supports could offer” (EDI-010)*.

For one respondent this apparent lack of engagement by CAMHS was seen as being unwelcome or inappropriate:

“*I have known CAMHS drop out because they've disclosed something, which is shocking” (MAN-015)*.

#### Subtheme 3: A question of trust

Working with CYP referred to services because of OCSA, or where the abuse had been disclosed during therapy, was seen to be dependent on CYP being able to trust the professional they were working with and for the service to provide a safe space to talk:

“*I suppose it's just, to giving a space to, to make it ok to talk about it, because I think the guilt and shame often for a lot of young people when it comes out that they've been sending pictures, erm, is that, and I try to keep as ordinary as possible if that makes sense, so I suppose the model is just to, to give, to hopefully allow a space where they can talk about things and feel they can be open about it, and not be judged” (ED-007)*.

Across the interviews and focus group reference was made to the shame experienced by young people and the impact that this may have on the ability to disclose what had happened, “*a lot of people don't obviously tell people, because of the shame and embarrassment” (ED-009)*. Where services deliver support anonymously, this can have a positive impact on young people's ability to talk about their experiences:

“*…it feels a bit safer to ask this rather than their parents, because that's obviously then a fear of how their parents will react, so I guess with coming to us, it just feels like a really safe space to talk about it without any consequence as such” (MAN-011)*.

Trust was not seen by respondents as something that could be taken for granted in cases of OCSA, as central to the experience was very often a perceived breach of trust:

“*…especially if they've experienced online sexual exploitation, I guess, that is really kind of, you, it might affect someone's trust in other people and affect kind of their other relationships, so, it might be trying, it might help them to kind of recalibrate who they can trust and, and, and notice you know what other people's intentions are, because they might be skewed after experiencing something like that” (ED-010)*.

However, building trust often had to be balanced with safeguarding procedures and being clear with the young person about some of the potential consequences of disclosure:

“*Whereas for other practitioners, or in other settings, or sometimes for me as well too, there's a real tension between safeguarding and getting alongside, because when you, when you're trying to implement safeguarding, you have to kind of, you might have to be quite firm and quite kind of procedural, and put things in place that feel very restrictive to the young person, and that's a necessity, but it can also make building trust quite difficult. If that makes sense?” (MAN-006)*.

#### Subtheme 4: Something new to grapple with

Some practitioners saw OCSA as one aspect of sexual offending and therefore felt that there was an existing framework to work within:

“*Erm, and actually most of the people that I'm thinking of, who I know have, sort of, have been, erm, victims of this really, have themselves experienced sexual abuse as a child” (MAN-014)*.

However, others described OCSA as something that had aspects that were different from contact sexual offending, and where boundaries around what was safe or acceptable behavior no longer existed:

“*you can think alright we get more and more sophisticated with our technology to kind of circumvent that or kind of triangulate all these pieces of data and pull it altogether and do an analysis of that, but then they also, and I suppose it's the bit of, you don't want to frighten young people but you need to give them those kind of basic messages about safety and personal safety in a, in a world where it feels that a lot of the time there are actually no boundaries whatsoever” (MAN-09)*.

Practitioners also saw that there were gaps in their own technical knowledge and understanding which left them unsure about how they could ask young people about their experiences:

“*Like I said, you know, er, a 4, my, my 4-year-old niece knows how to use the internet better than me” (MAN-015)*.

This sense of not having a shared understanding or a common framework of technology use was seen as problematic when working with young people:

“*I suppose it's a generational thing as well, is that some, er, sort of my generation, kind of a lot of the online world is unusual to us and we, it's confusing and, and kind of like, how does that work, erm, so understanding that, erm, so I think a lot, a lot of parents and, and professionals kind of don't use social, I mean might use Facebook maybe, but not many people are either the using the, the communication systems that young people use as well, so it's the dynamics of those, and, just understanding what's happened” (MAN-012)*.

For some practitioners this lack of a common framework left them challenging whether this was a “new normal” that they were now having to deal with:

“*So I'm aware of some apps, some really explicit apps that young people tell me about, but it's dropped in the conversation, like it's what everyone does, but actually those apps are explicit and they're of private areas, but everyone's got them, so everyone's doing it and you're almost sucked into think, ok, is this the new norm, not that it's ok, but, it's, it's minimised, you know, in that peer group, so they themselves aren't aware of what's happening is actually wrong or exploitative, or is abuse sometimes as well, you find yourself sort of thinking over it, is this me, or is this a world that I'm just not aware of” (EDI-FG1-006)*.

While explicit reference was made to some applications that are used by young people, this was done in a way that seemed to reflect a wide gap between themselves and the young people they worked with:

“*and I suppose for online, again maybe I can't imagine, but, it, it's the whole, like when I think online, I think, online and the internet but there's like apps and Snapchat and yeah, but it depends whether people are meaning, through kind of social apps as well as, do you know what I mean?” (ED-009)*.

There was also an appreciation that use of the internet was for most young people embedded into many of their daily activities such that limiting access—which might have been a traditional way of safeguarding children from sexual abuse—was no longer such a viable option:

“*it's just so hidden and difficult to, to, erm, to block out of their life, because young people are, they, they, they rely on social media so much that the, you know, and this won't be a new thought will it but to shut down social media for a young person is to deprive them of their entire social network, and so that doesn't seem like a very feasible option in terms of, of dealing with, erm, the abusers access to them*” (*MAN-001*).

###  Theme 3: It's emotionally charged

#### Subtheme 1: Impact on the practitioner

One unexpected finding from the analysis was the negative emotional impact of working with cases involving OCSA. This was evident in the language used across the interviews and focus group where adjectives such as “awful,” “horrendous,” and “horrible” were frequently used. Some of these cases were described as “never-to-be-forgotten” and raised issues as to what made OCSA so different from the cases of sexual abuse that practitioners frequently worked with:

“*one that stands out that I think I'll never forget, erm, was a young, a young girl… they did send her money, erm, and she'd sent videos of herself, all sorts, you know, naked pictures, videos, really explicit, horrible, I don't, I don't want to get into too much detail… things that were just disgusting, that they were making her do and, and then, erm, saying that this, threatened her basically, saying that, erm, they'd end up, they'd tell her, ‘cos they knew what school she went to, tell all the school, they'd tell mum, and she was, oh, just like, mortified” (MAN-004)*.

On occasions cases involved multiple perpetrators engaging in sexually abusive practices. This seemed particularly overwhelming for one practitioner:

“*But it wasn't just by one person… there were times when we looked through the phone and there would be, you know, sometimes 40 different guys, saying, hey, hit me up, blah, blah, blah, you know, do you want some of this, do you want some of that, erm, it was just endless” (MAN-005)*.

Practitioners described how they had to stay in control of themselves and not show distress at what the young person was saying. This was seen as an important skill and part of being “professional”:

“*it was making sure that like we're trained to, not, you know, if someone discloses something that about online sexual abuse or sexual abuse, we, we're not like, the, we, I don't know, it's about knowing how to compose yourself so, you can't look upset, even though sometimes I've wanted to cry, some of the things I've heard, you can't, you've gotta be professional, compose yourself, not acted shocked, and not act disgusted or, so, again, I feel like it's important skill” (MAN-004)*.

Listening to accounts of OCSA was associated with not only a distress response toward the particular CYP whom the practitioner was working with, and which could persist long after the case was closed, but also a reminder that this type of abuse might happen to a young person who was close to the practitioner:

“*I'll never, I'll always remember that for some reason, ‘cos of how awful it was, some of the things that they were making her do and, and the threat, you know, how severe it got, and, it's hard sometimes to switch off and, and forget that, and I've got a younger brother and sister who, who are teenagers, and it makes you like, a little bit like, hypervigilant you know, like with their, their, er, phone use and just trying to make them aware of, of what, you know, not trying to like lecture them, but it does it, it really, it impacts you personally as well” (MAN-004)*.

In some instances, feelings of distress within the team of practitioners warranted an organizational response to support staff. In the following extract, this followed when there had been a disappointing outcome to a case:

“*And I think ultimately it is that bit of, so we had to do a lot of kind of therapeutic work in a sense with our team… we had a team that had worked very closely with a young person that had come in, made the disclosure, was with us for nearly two years… CPS were involved and then turned round and said they weren't kind of pursuing the case and that team and that feeling of, they'd done, not that, well in a sense, yeah, but not that they'd done something wrong, but they'd failed, and when they'd worked so hard with somebody, really intensely, to then kind of, sort of like, take on, I've failed here or I've done something wrong…” (MAN-009)*.

#### Subtheme 2: Reflecting on own experiences

The responses of practitioners to OCSA were not all negative and clearly in some instances they could relate to what young people had done:

“*I would have definitely sent images of myself to a girl, definitely*” (*MAN-012*).

However, for some practitioners there was not only acknowledgment of potential risk-taking, but also reference to their own earlier vulnerabilities:

“*but if I'm thinking back to when I was younger, erm, and very insecure, particularly at high school age, I would say that maybe someone showed me they, they were interested in me, in, in any way not necessarily in a romantic way, I think I could have possibly, be putting myself, er, in a position that may, may have been risky… I think that might, you know, might make you feel very tempted and, and sadly online that's what these groomers may be praying upon, so, yeah” (MAN-07)*.

Also highlighted was the struggle to relate to what young people may see as “normal activity.” This again was positioned as a generation gap between practitioner and client*:*

“*there's a normalising of sending pics as well just now that's really difficult I think for myself as somebody who's older to get my head round” (ED-007)*.

#### Subtheme 3: Involving caregivers

Practitioners working with OCSA had to stay within existing organizational procedures and practices and while this varied across organizations, this inevitably involved decisions about the involvement of care givers:

“*the other thing I suppose to bear in mind is because we are in a young persons unit, there's obviously, there's very little negotiation around what happens once people do disclose that, there's obviously processes we have to follow, including, obviously, sharing that risk and sharing that knowledge with parents” (MAN-005)*.

Where cases involved abuse through, for example, image creation by a parental figure, safeguarding procedures were seen as similar to those involving contact offenses, but there were then also issues as to what should be disclosed to the child (and potentially the non-offending carer). The following extract suggests that the young person was informed that images had been taken of the abuse and that this was associated with considerable distress:

“*but that young person had no knowledge that that was what was done, so that whole, but that was then how it was found out that that's what happened to them, so then the sort of catastrophic realisation that this act had been shared who knows where, erm, which I guess is, you know, again that all the sort of powerlessness, and exposure, it's just horrendous” (ED-FG1-006)*.

This was also seen in the following extract where a young person had been exposed to content that was highly problematic, and this represented a significant challenge for practitioners:

“*Yeah, you can't un, you can't unsee something, so, if we're dealing with a child who's witness to something that's far beyond their understanding and, and they're development and cognitive ability, they've seen it, so how do we help them cope with that?” (MAN-012)*.

However, carer involvement was seen as an important part of how cases could be managed and as a way of working collaboratively to protect young people from future risks:

“*Erm, but possibly also thinking about practical things, you know, limits, or you know helping families think about what, should we be all be on phones in the middle of the night, you know I was working with a family and actually it took ages, but eventually they were like, you don't actually need to be on your phone in the middle of the night, you know we can all, the Wi-Fi can go off at night and actually that just reduced quite a lot of what was happening, because, they couldn't get access to the internet at night, you know it was quite simple, but it helped to sort of bring some of the distress, erm, down a little bit…” (ED-FG1-006)*.

Practitioners also talked about family homes that were chaotic with few boundaries around technology use. One of the overriding problems in relation to caregiver involvement was again a perceived lack of understanding of what young people were doing online or a minimization of the seriousness of some of the risks that were being taken:

“*parents where often, they don't have a clue what young people are [doing] online anyway, and then, erm, the kind of the difficulty we have is where people have been groomed quite prolifically online, it's affected their parents often seem quite unable to put the methods in place which mean that the young person's often back doing, kind of… or back involved with the people before, erm, so that's quite tricky I think” (ED-009)*.

This meant that working with carers often involved educating them about online risks and supporting them to learn to trust and engage with their children. This was often in the context of practitioners feeling a lack of confidence in their own knowledge base:

“*they just have no idea because they don't use online platforms, erm, so it's about educating the, the parents as well, and very sensitively explaining that maybe they should, check a bit more where, and, and how their young, their child is using, erm, online platforms, gaming, whatever…” (MAN-007)*.

Anxieties were also expressed that when carers were informed about what had happened to the CYP, practitioners' distress was amplified by the response of the carers:

“*the anxiety that the parents have of getting to know the young person has been abused, no matter what kind of abuse, can be worse than actually the young person's anxiety, you know, and it's, it's like, it just makes, it just amplifies everything for the young person” (MAN-002)*.

For this practitioner, the revelation to the family of what had happened was vividly expressed as “it just makes the whole volcano explode.” Added to this was that practitioners had to manage disclosure to parents about events that the young person was ashamed of and wanted to be kept secret:

“*The only one thing that person didn't want me to do was to tell the parents, because they felt so, so bad about what they'd done” (MAN-007)*.

## Discussion

Our results, particularly themes one (Breadth of the problem) and two (Working with OCSA), are similar to those found in other studies (33, 38–43) in that practitioners acknowledged the growing number of CYP whose presentation included OCSA, but there were differences in how this was conceptualized, the importance they placed on it, and their responses to either a disclosure by the CYP or information provided by an earlier assessment and included in the referral process. In particular, there was a blurred boundary between practitioners who described discrete manifestations of OCSA and those who used a more inclusive definition of sexual crimes involving CYP that were enabled by technology. There was a heightened awareness by practitioners of the role that sexual images played in OCSA, and there were particular concerns about the problems of First Person Produced Images (FPPI, or sexts) and peer related sexual activity. This led to confusion about how to position the consensual sharing of sexual images and their non-consensual distribution to other CYP. These concerns have been noted in another practitioner study which explored focus group data from Internet child exploitation law enforcement, child protection, and children's mental health practitioners (53). Their respondents struggled to see where FPPI cases would fit into their existing professional framework. As with the current study, there were concerns about CYP and sexual agency, the dangers of rapid technological change, and how this reinforced perceived generational differences. This led practitioners to question whether some behaviors were the “new normal,” which was something they felt particularly uncomfortable with. In a similar way, a generational gap between CYP and their parents, teachers, and law enforcement in relation to online risk-taking, managing feelings of fear and shame, and lack of information about disengaging from, and resolving, problematic relations where technology has played a part has previously been noted (54). In the UK, the change of terminology from self-produced sexual images, or sexts, to FPPI reflects concerns around the potential for blame to be placed on CYP where sexual images have been shared. Certainly, practitioners within the study appeared to feel some ambivalence about how to position children who had been victimized, but who had also victimized others. This was not the case where an adult was identified as perpetrator and there was clear evidence of a power imbalance.

What was apparent in our results was that many practitioners did not directly ask questions about OCSA and either waited for the CYP to mention this or indirectly approached the topic through talking about technology use or adolescent friendships and relationships. It was noted that they had no assessment tools to help with this and none of the practitioners in our sample made mention of staff training. This meant that they drew on existing practices, some trauma focused and others relational approaches, when working with these CYP, similar to findings from other studies (41, 55). Reference was made to existing research on OCSA and digital safety, which was shared with caregivers as part of the intervention, but there was a marked lack of confidence in whether practitioners had sufficient expertise in relation to this. Other studies have noted this (42) and how this lack of confidence influenced the willingness to inquire about digital risk-taking unless there were very specific issues around safeguarding. In these circumstances, the fall back was to use standardized safeguarding approaches. What was also noted by one practitioner in the current study was the absence of referral pathways and this, in the context of a lack of training, left people struggling to find a best fit for these cases, or how to source other agencies to refer CYP onto. Organizational barriers created a perception by staff that OCSA was not seen as a priority with these children's mental health services, and that this was also shared with other agencies such as police and social work.

Practitioners identified that OCSA was associated with high levels of shame and self-blame in CYP, and that central to any intervention was the ability by the practitioner to establish a trusting relationship and the need to provide a safe space. It was felt that this was potentially compromised by practitioners having to comply with safeguarding procedures which may involve contacting the police or social services or the removal of a hand-held digital device to ensure no further contact could be made with a perpetrator. It was felt that for CYP, ambivalence about what may be seen as acceptable relationships and appropriate behavior online may make disclosure of OCSA challenging. Issues were also raised about how trust could be established with practitioners where there was limited shared understanding about what had happened, the technologies that had been used, and the need of the CYP to try and maintain control over what had happened. Many studies have noted the reluctance of CYP to disclose their experiences of OCSA (35), and in one of the first studies in this area most children did not disclose, and even when confronted with the photographic evidence of the abuse, they would only acknowledge what they thought was already known (33). This would seem to suggest that only a few children who have experienced OCSA will be identified, and even fewer will be provided with meaningful therapeutic support.

Many practitioners identified OCSA as providing new scenarios that they had to manage, and as with other studies, there was a lack of clarity about whether (and when) CYP required support, or possibly the involvement of other agencies, such as law enforcement (53). Across the interviews and focus group there was evidence of ambivalent perspectives about OCSA, particularly in relation to the involvement of peers and this moved between a permissive to a more punitive stance. However, as seen in the findings related to Theme 3, these new case scenarios had a considerable impact on practitioners, which was reflected in the language used to describe them. It was not clear from practitioner accounts whether they were exposed to CSAM when working with CYP, but this seemed to be the case for some, and our findings resonated with distress reported in specialist police groups (56) and the potential need for organizational support for staff (57) as well as further training. For some, disclosure of OCSA by CYP triggered practitioner anxieties as to whether children within their own family or social network might have been subject to online harm.

Practitioners had to make decisions about the involvement of caregivers in cases where OCSA had been disclosed. This posed challenges, especially where OCSA was not the primary reason for referral, the caregivers were not aware of the abuse and the CYP did not want their caregivers to know. In some instances, children had been sexually abused and were unaware that the abuse had been photographed and the images uploaded. Practitioners were then faced with making a decision about informing the child, which involved balancing an assumption about the rights of the child to know what had happened alongside their right not to be told (58). What was noted in our data was the added distress experienced by CYP when informed about the presence of sexual images and the amplification of distress when this involved caregivers (59).

## Strengths and limitations

We used a qualitative design with a reflexive thematic analytical approach to analysis. A sample of 25 is in keeping with this method and, while we were cognizant of a tool for determining sample sizes in thematic analysis research (60), instead we chose to follow the argument presented by Braun and Clarke (45). Participants included those who identified as male/female, but there were more women than men and there was low ethnic diversity. Both issues need addressing in future research, particularly as there may be implications for disclosure. The sample was recruited from two sites. We do not know whether this influenced the types of cases that participants were exposed to; although, this is likely in relation to CAMHS. A larger sample size may have allowed us to examine this in our analysis.

## Conclusion

This qualitative study indicated that, within this sample of practitioners working with CYP, there is a growing awareness of OCSA both as a presenting problem within services as well as co-occurring with other mental or sexual health problems. Most practitioners did not directly ask questions about OCSA and felt that often they were reliant on CYP disclosing what they had experienced. The implication of this was that practitioners had to draw on existing practices in relation to assessment and intervention and it was observed that they had few evidence-based tools or strategies to draw upon, and little direct support from within their organizations. This not only had relevance for their work with CYP, but also in relation to practitioner well-being and support. Exposure to these forms of abuse seemed particularly distressing and led to reflections on what might be happening to CYP within their own social and familial contexts. Within the interviews, there was evidence that practitioners acknowledged the centrality of technology in CYP's lives. We would argue that frameworks that help conceptualize and assess this as part of the ecology of the CYP may have great utility (16, 17). What was also evident were anxieties about potential gaps between practitioners and CYP's knowledge and use of technology and its applications. This requires attention, as anxieties about understanding the technological affordances offered to CYP and their association with OCSA will impact the discovery of harms that have been experienced alongside future vulnerabilities (61).

## Data availability statement

Due to the sensitive nature of the topic of this study and consent procedures used for data sharing, the data is not suitable for sharing. Requests to access the datasets should be directed to sandra.bucci@manchester.ac.uk.

## Ethics statement

The studies involving human participants were reviewed and approved by Research Ethics Committee 4, West of Scotland. The patients/participants provided their written informed consent to participate in this study. Written informed consent was obtained from the individual(s) for the publication of any potentially identifiable images or data included in this article.

## Author contributions

SB, EQ, MS, FV, and KC conceptualized the study and supervised data collection. WH, CC, and AN collected the data. EQ and SB wrote the first draft of the paper. All authors were involved in developing the analytical framework, and reviewed the paper. All authors contributed to the article and approved the submitted version.
